# Horizontal inequity in the utilisation of Continuum of Maternal Health care Services (CMHS) in India: an investigation of ten years of National Rural Health Mission (NRHM)

**DOI:** 10.1186/s12939-021-01602-3

**Published:** 2022-01-15

**Authors:** Sumirtha Gandhi, Umakant Dash, M. Suresh Babu

**Affiliations:** 1 Bengaluru Dr. B.R. Ambedkar School of Economics, Bengaluru, Karnataka India; 2 Institute of Rural Management, Anand, Gujarat India; 3 Department of Humanities and Social Sciences, Indian Institute of Technology, Chennai, India

**Keywords:** Continuum of maternal health care services, National Rural Health Mission, Horizontal inequity, Erreygers corrected concentration indices

## Abstract

**Background:**

Continuum of Maternal Health Care Services (CMHS) has garnered attention in recent times and reducing socio-economic disparity and geographical variations in its utilisation becomes crucial from an egalitarian perspective. In this study, we estimate inequity in the utilisation of CMHS in India between 2005 and 06 and 2015-16.

**Methods:**

We used two rounds of National Family Health Survey (NFHS) - 2005-06 and 2015-16 encompassing a sample size of 34,560 and 178,857 pregnant women respectively. The magnitude of horizontal inequities (HI) in the utilisation of CMHS was captured by adopting the Erreygers Corrected Concentration indices method. Need-based standardisation was conducted to disentangle the variations in the utilisation of CMHS across different wealth quintiles and state groups.  Further, a decomposition analysis was undertaken to enumerate the contribution of legitimate and illegitimate factors towards health inequity.

**Results:**

The study indicates that the pro-rich inequity in the utilisation of CMHS has increased by around 2 percentage points since the implementation of National Rural Health Mission (NRHM), where illegitimate factors are dominant. Decomposition analysis reveals that the contribution of access related barriers plummeted in the considered period of time. The results also indicate that mother’s education and access to media continue to remain major contributors of pro-rich inequity in India. Considering, regional variations, it is found that the percentage of pro-rich inequity in high focus group states increased by around 3% between 2005 and 06 and 2015-16. The performance of southern states of India is commendable.

**Conclusions:**

Our study concludes that there exists a pro-rich inequity in the utilisation of CMHS with marked variations across state boundaries. The pro-rich inequity in India has increased between 2005 and 06 and high focus group states suffered predominantly. Decentralisation of healthcare policies and  granting greater power to the states might lead to equitable distribution of CMHS.

**Supplementary Information:**

The online version contains supplementary material available at 10.1186/s12939-021-01602-3.

## Introduction

Concern for equity imbibes a positive spirit and any systematic deprivations resulting in poor maternal and child health outcomes should be eliminated from the society. A transformative shift from the Millennium Development Goals (MDGs) to the  overarching Sustainable Development Goals (SDGs) underscores the need to address maternal health issues in a more innovative manner [1]. Studies on the utilisation of maternal health care services have highlighted an abysmally poor condition of maternal health in developing countries including India. Around 15% of the world’s maternal deaths are contributed by India [2]. Although Maternal Mortality Rates (MMR) in India fell from 556 (1990) to 113 (2016-18) [3], the progress was widely different across  states . For instance, the MMR in high focus group states fell from 520 in 1997-98 to 375 in 2004-05 and 161 in 2016-18 [4, 5]. Whereas in the southern states, the corresponding estimates were much lesser, ranging from 187 (1997-98) to 149 (2004-05), and 67 (2016-18) [4, 5]. The high MMRs are primarily attributed to the negligence of continuum of maternal health care services (CMHS) [6–8]. CMHS recognises a need to undertake maternal health care services throughout the cycle of pregnancy and motherhood, including full antenatal care services (ANC), delivery under the supervision of skilled birth attendant (SBA) and post-natal care services (PNC) [9]. The argument is that utilisation of these services is intertwined with each other and yields better health outcomes when consumed in a continuous/sequential manner [10]. Existing studies also highlight that the utilisation of CMHS can reduce MMR by 15% [11].

One of the major concerns of India’s health system is the iniquitous distribution of maternal health care services across the states and income quintiles [10, 12, 13]. The prevalence of socio-economic and geographical inequalities in the utilisation of health care services led to the culmination of National Rural Health Mission (NRHM) in 2005-06, later renamed as National Health Mission (NHM) in 2013 [14]. The implementation of NRHM is rooted in an egalitarian framework and  functions with two important objectives- First, reduction of maternal and child deaths by promoting utilisation of CMHS; and second, curtailing disparities in the utilisation of CMHS across socio-economic and geographical groups. The NRHM  used a set of crucial strategies such as increasing public health funding, decentralisation of health planning at village and district levels, promoting social and community participation and strengthening community empowerment [15, 16]. Supply strengthening interventions such as employing Accredited Social Health Activists (ASHA) and demand side financing were also implemented to increase utilisation of maternal health care services among poor women [15]. The implementation of NRHM varied across high-focus^1^ and non-high focus group states.^2^ The categorisation of these groups was determined by the performance of maternal health indicators. NRHM was initially rolled out in high focus group states which are considered as deprived/ less developed states of India - these states were entitled for higher financial, technical, and managerial assistance from the central government [15].

Studies investigating the utilisation of maternal health care services in the pre and post NRHM period [16–19] indicate that the there was consistent increase in the utilisation of ANC, SBA and PNC. Few of them highlight a reduction of rich-poor gap in the utilisation of delivery and post-delivery care after the implementation of NRHM [17–19]. Others highlight that the implementation of NRHM favoured high focus group state s[17]. Some studies have conducted inequality analysis by either encompassing both need and non-need based indicators together [17, 19] or by taking individual maternal health interventions [19]. To our knowledge, inequity analysis using a comprehensive maternal health interventions are scant in the literature. Such insights are crucial but do not indicate the extent of inequality caused by illegitimate indicators^3^ or non-need based factors which is ethically unacceptable. Hence, there are two important gaps in the literature. First, standardisation of need-based factors is crucial to understand the extent of inequity because any variation in the distribution caused by a need-based factor (biological need) doesn’t reflect an unfair event which is ethically unacceptable. On the other hand, variations caused by illegitimate factors such as social determinants are dangerous from an egalitarian perspective and hence ending them becomes a major distributional concern for policy makers and health system. Second, to illuminate disparity in CMHS, the definition of CMHS must be followed in a more structured manner.

In this regard, our study makes a novel contribution in the realm of equity based research in maternal health. In this study, we attempt to understand the horizontal inequity in the utilisation of CMHS in India as a whole, as well as across Indian states between 2005 and 06 and 2015-16. To define the utilisation of CMHS, we have considered those seeking neither ANC, SBA or PNC or any one of the services as ‘not seeking CMHS’ and those seeking ANC, SBA and PNC as ‘seeking CMHS’. Researchers suggest that the measure of horizontal equity is not possible without defining vertical equity [20]. Vertical equity is the unequal treatment for unequals and Horizontal equity requires  that the distribution of resources be apportioned according to needs [21]. To compute horizontal inequity, we have adopted the Erreygers corrected concentration index [22] which satisfies all four basic assumptions of rank dependent indices to measure horizontal inequity as against the standardised concentration method [23–25] which satisfies only one assumption. Moreover, Erreygers method is considered most suitable when health variable is bounded and ordinal in nature – as in our study. Finally, we employ decomposition analysis technique to enumerate the contribution of individual level covariates on inequity in the utilisation of CMHS.

## Empirical framework

### Data sources

**Fig. 1:**
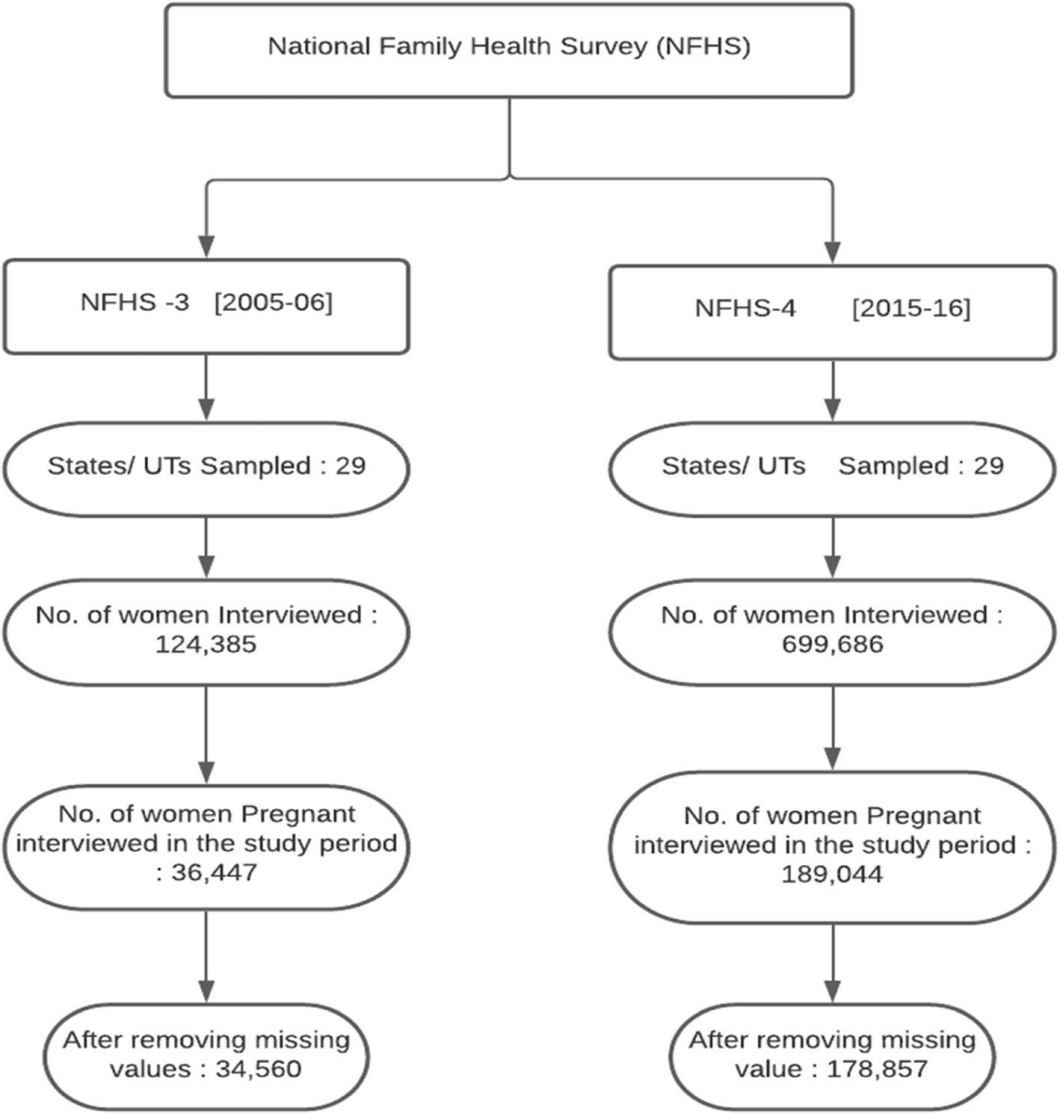
Study Flow Chart

Individual level cross-sectional dataset was extracted from the two rounds of NFHS conducted in 2005-06 and 2015-16. NFHS is carried out at regular intervals in India under the stewardship of the Ministry of Health and Family Welfare and Institute of Population Sciences (IIPS). The former is representative at the state level, while the latter renders information at district level. In both the rounds of NFHS, stratified random sampling design was adopted for sampling. In rural areas, two stage sampling procedure was followed, where primary sampling units, i.e., or villages, were selected through probability proportional to population size (PPS) and households were selected using equal probability approach. In the urban areas, sampling was conducted in three stages. In the first stage, wards were selected using probability population size. In the second stage, census enumeration blocks (CEB) were selected from each of the chosen wards through PPS. Finally, in the third stage, households were randomly selected from each of the CEB. A total of 699,686 (NFHS - 4) and 124,385 (NFHS-3) women belonging to an age group of 15–49 years were successfully interviewed. After removing the missing values, we arrived at a final sample of 34,560 in 2005-06 and 178,857 in 2015-16. To ensure consistency across the two rounds, we have removed information of Union territories from NFHS-4. The details are provided in Fig. 1.

### Selection of variables

Outcome variable is a binary variable coded as 1 (if all three maternal health services-ANC, SBA and PNC- are undertaken) and 0 (if any  or none of the three maternal health services are undertaken). In this case, 1 represents CMHS and 0 represents partial/no care. Explanatory variables of this study are broadly categorised into legitimate/need based factors and illegitimate or non-need based factors. Although need is an elusive concept, we have chosen the most appropriate indicators to represent the need of pregnant women. According to the literature, Body Mass Index (BMI) of a pregnant woman, birth order and age of a pregnant woman can be used as a proxy for need based indicators [26]. The set of non-need based indicators considered in this study are mother’s education, caste, religion, residence, access to media exposure, barriers related to access^4^ and community level education.^5^

### Empirical methodology

The standard concentration index (CI) has been extensively used for the calculation of health inequality [23–25]. This can be computed as follows:

1 $$\kern4.5em CI=\frac{2}{\overline{h}}\mathit{\operatorname{cov}}\left({h}_i,{y}_i\right)$$

Where, *h*_*i*_, is the health condition of individual i, *y*_*i*_ is the socio-economic rank of an individual (*i*) and $\overline{h}$ is mean health status of the entire population. The CI is twice the area between the concentration curve and line of equality (45-degree line). The value of CI ranges between − 1 and + 1, where a positive value indicates pro-rich distribution and a negative value represents pro-poor distribution.

However, when the outcome variable is binary in nature, the application of concentration index approach might provide flawed estimates [22]. This approach has certain setbacks. First, these bounds ranges between $\overline{h}$-1 and 1- $\overline{h}$, where $\overline{h},$ the mean of the outcome, thereby limits the measurement of is socioeconomic related inequalities in health. Second, CI ranks countries by inequalities in health and ill-health differently [27]. Third, the maximum and minimum value of CI depends on the mean of the health outcome in the society (Erreygers, 2009). Lastly, the value of CI depends on the scale of the health variable and might produce flawed estimates when the health variable is binary. To resolve these issues, Wagstaff proposed a corrected version of CI accounting for the feasible bounds of the CI for a binary variable. It is calculated by dividing the standard CI formulae by $\left(1-\overline{h}\right)$

2 $$\kern2.75em W=\frac{1}{\overline{h}\left(1-\overline{h}\right)}2\ \mathit{\operatorname{cov}}\left({h}_i,{y}_i\right)$$

This approach was criticized by Erreygers (2009) mainly because *(i)* it normalizes CI arbitrarily (Erreygers, 2009);(*ii)* it does not measure absolute or relative inequality [28], and *(iii)* it is not invariant to equal treatments in health. Erreygers Corrected Concentration Index Method [22] is an alternative normalization technique measuring absolute inequalities in health. It is computed using the following Eq. (3):

3 $$EI={f}^E\left({\mu}_h,n\right){\sum}_{i=1}^n{z}_i{h}_i=\frac{8}{n^2}{\sum}_{i=1}^n{z}_i{h}_i$$

Where ${z}_i=\frac{n+1}{2}-{\lambda}_i,f\left({\mu}_h,n\right)>0,n$ is the number of individuals belonging to a given population, and *λ*_*i*_ denotes the socioeconomic rank of an individual ranging from richest (*λ*_*i*_ = 1) to poorest (*λ*_*i*_ = *n*); *h*_*i*_ is the vector of binary health variable while *μ*_*h*_ represents the mean health status of the total population.

We can also express Erreygers Index (EI) in the following algebraic form:

4 $$EI=\frac{4}{b_H-{a}_H}2\ \mathit{\operatorname{cov}}\left({h}_i,{y}_i\right)\kern1em or\kern1em EI=8\mathit{\operatorname{cov}}\left({h}_i,{r}_i\right)$$

Where *h*_*i*_ is the health variable of interest, *r*_*i*_ is the individual or respondent’s relative rank in the socioeconomic variable distribution. Here, the size of EI reflects the strength and variability in the health variable of interest. Positive (negative) values of EI indicate a pro-rich (pro-poor) distribution. One of the major advantages of this index is that it satisfies four essential criteria [22]. They are: 1) Transfer: A small transfer of the variable of interest from a richer to a poorer individual translates into a pro-poor change in the inequality index, 2) Mirror: The inequality index of the variable of interest, and the inequality index of the shortfall of the variable of interest should be mirror images of each other, 3) Level independence: An equal increment of the variable of interest for all individuals does not affect the inequality index and 4) Cardinal Invariance: A linear transformation of the variable of interest does not affect the value of the index. We followed this approach to demonstrate horizontal inequity in health. Additionally, we conducted a decomposition analysis to unravel the contribution of socio-economic covariates.

The formulation of the decomposition analysis is presented in Eq. (5) below, the explanation of which is provided via nonlinear modelling:

5 $$\kern3.75em {h}_i=G\left(\alpha +{\sum}_j{\beta}_j{x}_{ji}+{\sum}_k{\gamma}_k{z}_{ki}\right)+{\varepsilon}_i$$

Where G (.) will take the form of nonlinear model (for instance, Logit/Probit), *x*_*j*_ are the need-proxies and *z*_*k*_ represents the non-need control variables. If there were no z variables, then predicted values obtained from the model could be interpreted as need-expected utilisation. A linear approximation of the model can be estimated by estimating partial effects of the non-linear model [25. The linear approximation to the previous equation is given by:

6 $${h}_i={\alpha}^m+{\sum}_j{\beta}^m{x}_{ji}+{\sum}_k{\gamma}_k^m{z}_{ki}+{u}_i$$

Need predicted utilisation provides the estimate that would be expected given the distribution of need and it is expressed in the following Eq. (7):

7 $${\hat{h}}_i^x={\hat{\alpha}}^m+\sum_j{\hat{\beta}}_j^m{x}_{ji}+\sum_k{\hat{\gamma}}_k^m{\overline{z}}_k$$

Subsequently, indirect standardised utilisation can be understood using Eq. (8):

8 $$\tilde{h}_{i}^{IS}={h}_i-{\hat{h}}_i^x+\hat{\overline{h}}$$

Where $\hat{\overline{h\ }}$ is the mean predicted  utilisation with all variables at actual values; ${\hat{h}}_i^x$ is the need predicted utilisation and *h*_*i*_ represents the actual utilisation which refers to the healthcare utilisation of the respondent indicated in the household survey. Need-predicted healthcare utilisation is used to capture variation in healthcare utilisation predicted only by need-based factors. Contrary to this, need-standardised healthcare utilisation is used to capture the gap between actual healthcare utilisation and need-predicted healthcare utilisation.

By undertaking a decomposition analysis, we derive the contribution of individual covariates to socioeconomic related inequalities in health. We employed EI approach considering the binary nature of the health variable (dependent/outcome variable) instead of the standard concentration index (CI), and decomposition of the concentration index was multiplied by 4 to obtain EI.

9 $$\kern7em EI=4\left[{\sum}_j{\beta}_j{\mu}_{x_j}{C}_{x_j}+{\sum}_j{\gamma}_k{\mu}_{z_k}{C}_{zk}\right]$$

Where μ represents the mean; β and γ represents the coefficient of the variable *x* and *z*, respectively. CI represents the standard concentration index and horizontal inequity (HI) is obtained by subtracting the need contributions from the unstandardised HI.

## Results and findings

### Descriptive statistics

Table 1 provides the descriptive statistics of the covariates along with their mean and standard-deviation (SD) for 2005-06 and 2015-16. Age range  of sampled population was mainly 25–29 years in 2005-06 (Mean = 0.346; SD = 0.472) and 2015-16 (Mean = 0.368; SD = 0.482). Majority of women had birth order less than 4 in 2005-06 (Mean = 0.746; SD = 0.435) and it increased in 2015-16 (Mean = 0.822; SD = 0.383). Number of households with 6 members or less increased between 2005 and 06 (Mean = 0.589; SD=SD = 0.492) and 2015-16 (Mean = 0.618; SD = 0.486) indicating a fall in the household size. The mean value of the sampled population hailing from rural areas was around 0.75 in both 2005-06 and 2015-16. Age of marriage mostly ranged between 18 and 23 in 2005-06 (Mean = 0.418; SD = 0.493) and 2015-16 (Mean = 0.529; SD = 0.499). Further, women mainly belonged to Hindu religion in 2005-06 (Mean = 0.700; SD = 0.458) and 2015-16 (Mean: 0.729; SD = 0.444). Percentage of women possessing secondary education is found to be highest in 2005-06 (Mean = 0.384; SD = 0.486) and 2015-16 (Mean = 0.463; SD = 0.499).

**Table 1:** Descriptive Statistics

List of Covariates	2005–2006		2015–2016	
	Mean	Std. Dev	Mean	Std. Dev
***Non-need based factors/ illegitimate factors***				
***Household Size*** *(Ref: Greater than 6)*				
Less than 6 Members	0.589	0.492	0.618	0.486
***Number of Under-five*** *(Ref: Greater than 2)*				
Less than or equal to two children	0.129	0.335	0.441	0.497
***Residence*** *(Ref: Rural)*				
Urban	0.377	0.485	0.760	0.427
***Mother’s Education*** *(Ref: No Education)*				
Primary Education	0.144	0.351	0.141	0.348
Secondary Education	0.384	0.486	0.463	0.499
Higher Education	0.085	0.279	0.103	0.304
***Media Exposure*** *(Ref: No Media)*				
Access to at least 1 medium of information	0.635	0.482	0.646	0.478
***Caste***^***a***^ *(Ref: SC)*				
ST	0.158	0.364	0.193	0.395
OBC	0.336	0.472	0.392	0.488
Others	0.298	0.457	0.229	0.420
***Community Educational Status*** *(Ref: Low)*				
High	0.397	0.489	0.514	0.500
***Age at Marriage*** *(Ref: Less than 17)*				
18–23	0.418	0.493	0.529	0.499
24–34	0.092	0.289	0.114	0.318
Above 35	0.002	0.039	0.002	0.041
***Religion*** *(Ref: Muslim)*				
Hindu	0.700	0.458	0.729	0.444
Christian	0.094	0.292	0.075	0.263
Others	0.047	0.212	0.042	0.201
***Wealth Index*** *(Ref: Poorest)*				
Poor	0.181	0.385	0.232	0.422
Middle	0.203	0.402	0.201	0.401
Rich	0.219	0.414	0.171	0.376
Richest	0.222	0.415	0.145	0.352
***State Group*** *(Ref: Non-High Focus Group)*				
High Focus	0.634	0.481	0.755	0.430
***Access Related Barriers*** *(Ref: No barrier)*				
Acceptability	0.088	0.284	0.092	0.288
Availability	0.449	0.497	0.549	0.497
Affordability	0.075	0.263	0.074	0.263
***Need based factors/ legitimate factors***				
***Birth Order*** *(Ref: Equal or greater than 4)*				
Less than 4	0.746	0.435	0.822	0.383
***BMI Status*** *(Ref: Less than 18.5 & greater than 25)*				
Less than 18.5 and greater than 25	0.447	0.497	0.615	0.487
***Mother’s Age (Ref: Less than 20)***				
20–24	0.308	0.462	0.297	0.457
25–29	0.336	0.472	0.368	0.482
30–34	0.188	0.391	0.195	0.396
35–49	0.108	0.311	0.110	0.313
**Total**	**34,560**		**1,78,857**	

Source: Author’s Computation

^a^ Caste is defined as schedule caste (SC), schedule tribe (ST), other backward caste (OBC), and others. These terms are recognised in the constitution of India

The prevalence of community education was relatively low in 2005-06, which improved by around 10 percentage points through the decade. Caste-wise differentials asserted that most of the sampled population were from OBC category in 2005-06 (Mean = 0.336; SD = 0.472) and 2015-16 (Mean = 0.392; SD = 0.488). Majority of the pregnant women had access to at least one source of medium of information in 2005-06 (Mean = 0.635; SD = 0.482), which marginally increased in 2015-16 (Mean = 0.646; SD = 0.478). Region-wise estimates ascertained that, most of the women resided in high focus states in 2005-06 (Mean = 0.634; SD = 0.481) and 2015-16 (Mean = 0.755; SD = 0.430). The distribution of sampled women across wealth quintiles remained almost same; with majority of them belonging to the poorest quintile population. Most women faced barriers related to availability as compared to affordability and accessibility hinderances in both the time periods. The prevalence of those suffering from availability issues increased over time. In 2005-06, around 0.45 of women faced availability issues while accessing CMHS, and this number rose to around 0.55 in 2015-16.

### Mean of continuum of maternal healthcare: a comparison of standardized and unstandardized estimates

**Fig. 2:**
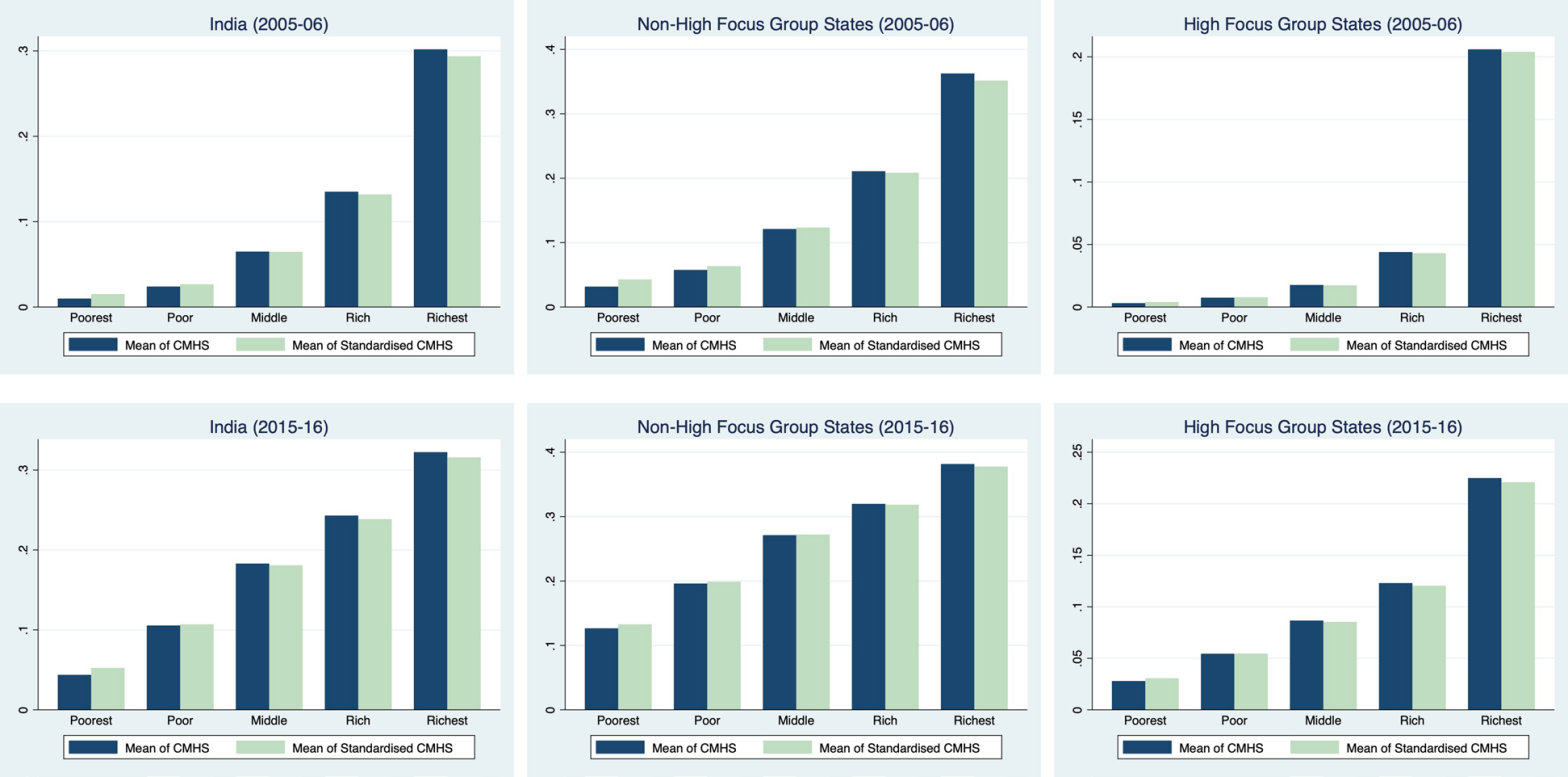
Mean of Continuum of Maternal Healthcare in 2005-06 and 2015-16: Standardised Vs. Unstandardised Estimates

Figure 2, compares standardized and unstandardized mean estimates of the utilization of CMHS across wealth quintiles and state groups. The differences between standardized and unstandardized mean values indicate the differences attributed by legitimate and illegitimate factors. A small difference between these two values suggests that the contribution of legitimate factors is marginal. Quintile-wise and state-wise estimation indicates that the differences between standardized and unstandardized values are quite low indicating a lower contribution of legitimate factors. However, the mean utilization of CMHS in both high and non-high focus states increased in the considered time period. However, the improvement was much higher for high-focus group states (4.5 percentage) compared to non-high focus group states (10 percentage points),  utilization was clearly higher among the rich/richest quintile population compared to the poor/poorer/ middle quintile population indicating an existence of pro-rich disparity in the utilization of CMHS. The pattern is quite noticeable for both 2005-06 and 2015-16.

**Fig. 3:**
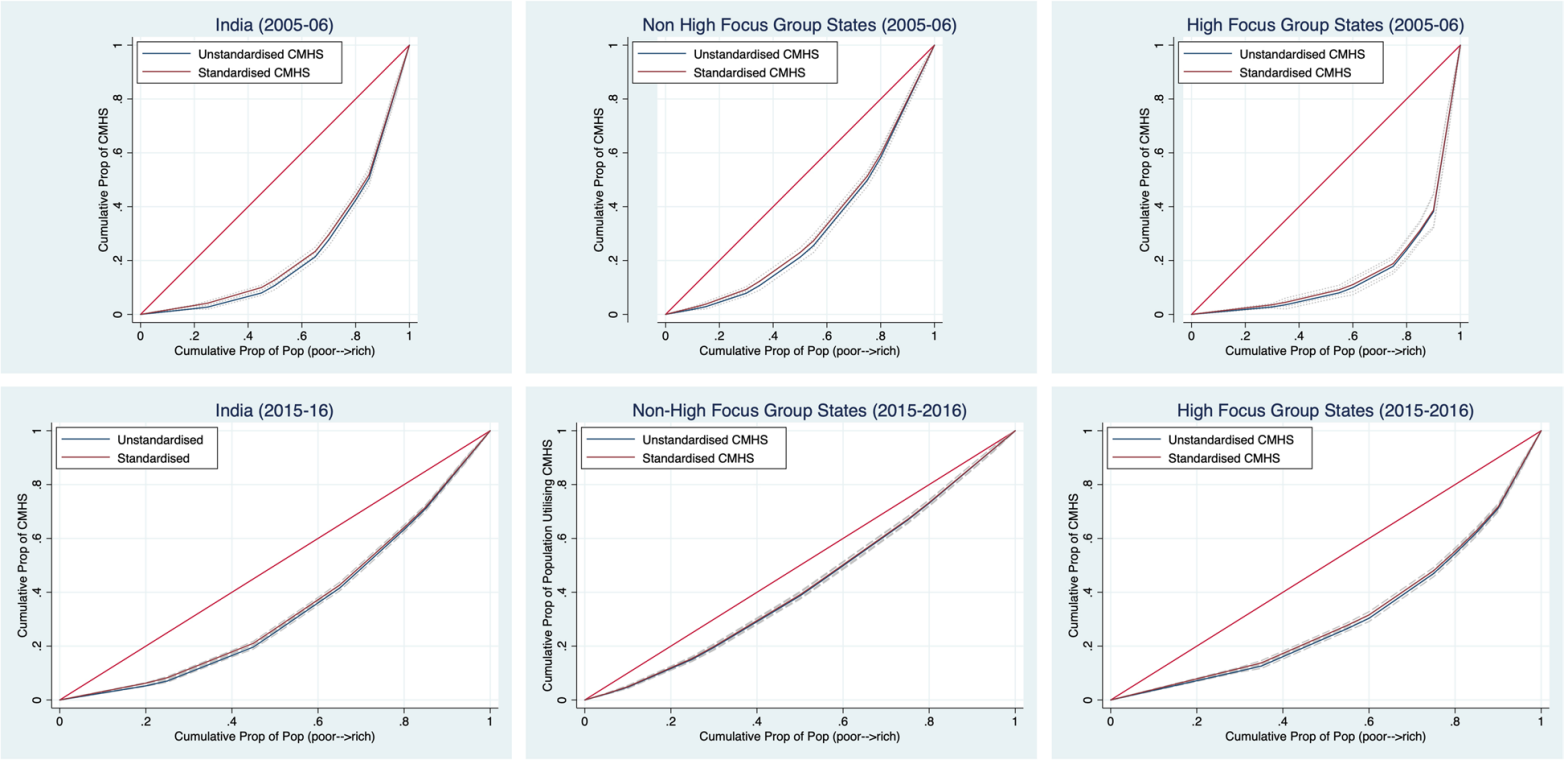
Concentration Curves of the utilization of CMHS across different regions in 2005-06 and 2015-16

Figure 3 captures the pattern of inequality using concentration curves (CC) disaggregated for high and non-high focus group states in 2005-06 and 2015-16. The concentration curves explain the relationship between the cumulative proportions of the population arranged from poorest to richest quintile group against the cumulative proportion of population arranged from lowest to highest utilization of CMHS. Here, the 45-degree line indicates the line of equality- when the CC falls towards the left, the distribution is comparatively more amongst poorer quintile population highlighting a pro-poor distribution, while a pro-rich distribution is witnessed when CC falls towards the right. The difference between the 45-degree line and the CC explains the magnitude of health disparity. Clearly, the gap between CC and 45-degree line is prominent across regions as well as time period. The gap is consistently higher in 2005-06 in comparison to 2015-16. The magnitude of the gap shrunk across the considered time period. The region-wise estimation indicates that in the non-high focus states, pro-rich inequality plummeted massively, while in high-focus grouped states, the level of pro-rich inequality was higher despite having witnessed a reduction between 2005 and 06 and 2015-16.

### Erreygers corrected concentration index

**Fig. 4:**
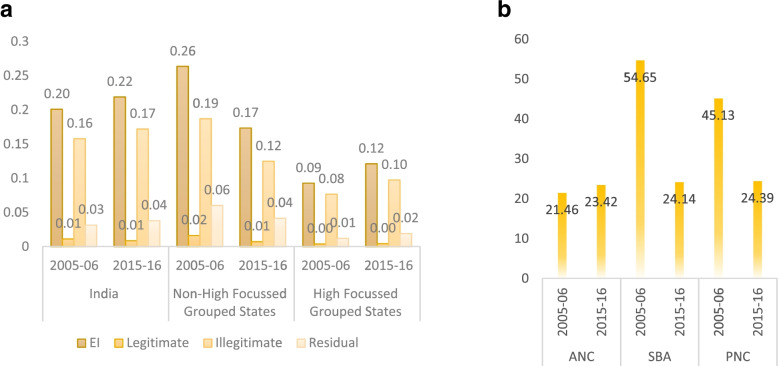
**a** & **b** Erreygers Corrected Concentration Index in 2005-06 and 2015-16: Across state-groups and individual maternal health interventions

The Erreygers Corrected Concentration Indices were constructed to discern the magnitude of HI after controlling for the influence of need based factors. The index value ranges between + 1 and − 1, where, a positive value signals an orientation towards pro-rich inequity, while a negative number suggests pro-poor inequity. Figure 4 (a) demonstrates the EI values disaggregated across state-groups, wherein the contribution of legitimate and illegitimate factor has been separately captured. Overall, the utilisation of continuum of maternal healthcare services is pro-rich in the considered period of time. Furthermore, the magnitude of pro-rich inequity increased by 1.2 percentage points between 2005 and 06 and 2015-16.

The level of pro-rich inequity has been compounded by a disparity in the high-focus group states. Factor wise disaggregation indicates a major contribution of illegitimate factors, thereby raising an alarming situation for the health system which is desperately fighting towards the reduction of inequity in the utilisation of CMHS due to variations in socio-economic determinants. In comparison to high-focus, the non-high focus group states witnessed a greater reduction in the pro-rich inequity in the considered period of time.

Figure 4 (b) highlights EI values for individual maternal health interventions and disentangles the variations in 2005-06 and 2015-16. The results provide some interesting insights with delivery (SBA) and post-delivery care services (PNC) witnessing a reduction in the pro-rich inequity. On the contrary, pre-delivery care services represented by ANC have shown an increase in the pro-rich inequity in the utilisation of CMHS (2005-06 vs 2015-16).

**Fig. 5:**
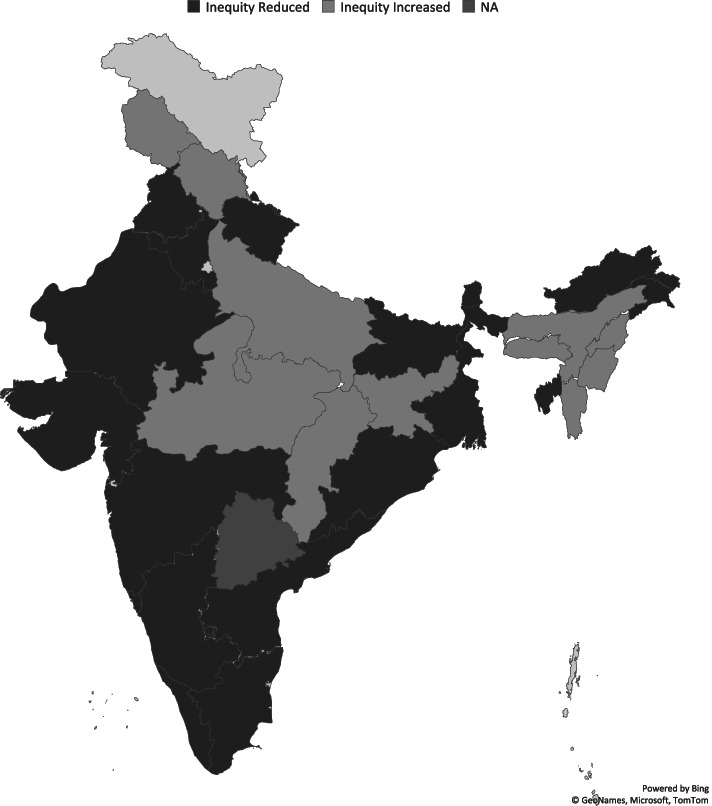
Change in Horizontal Inequity: Pre and Post Reform Period: State-wise estimation. Note: The details of each of the states are provided in Table (A.1) of Additional file 1

Figure 5 illustrates the state-level variation in the Erreygers Corrected Concentration Index (EI) for the utilization of CMHS in the Indian states between 2005 and 06 and 2015-16. As discussed earlier, the horizontal inequity for CMHS in India increased by around 1.08% between 2005 and 06 and 2015-16. The state-level estimation further reveals unconscionably wide disparities regarding the utilization of CMHS after the implementation of NRHM/NHM. Although inequity in utilization of CMHS at national level increased, we found that some states experienced a fall in the level of inequity, while others witnessing a rise in the considered time period.

### States recording a fall in the health inequity: a positive signaling

We found that the southern states (Tamil Nadu, Karnataka, Kerala and Andhra Pradesh) witnessed a reduction in the level of pro-rich inequity in the utilization of CMHS. The reduction was relatively higher in Karnataka (27.06%) and Tamil Nadu (22.4%) compared to Kerala and Andhra Pradesh. Other states which experienced a similar level of reduction are Sikkim (24.98%) from North-eastern region and Goa (23.99%) from the western part of India. Gujarat, Haryana, Bihar Maharashtra, Rajasthan, and Tripura were on the same trajectory – witnessing a reduction of pro-rich inequity by around 2 to 4 percentage points after 10 years of implementation of NRHM.

### States recording an increase in the health inequity: a worrying phenomenon

North-eastern states, namely Assam (10.58%), Meghalaya (13.11%), Mizoram (16.96%) and Manipur (29.97%) and Nagaland (3.5%) showed an increase in the level of wealth-based inequities in the utilization of CMHS. This indicates that NRHM was not able to improve the situation of most of these states- which were the primal focus of NRHM programs. A rise in the pro-rich inequity of around 3 to 6% was experienced by Himachal Pradesh, Jharkhand, Madhya Pradesh, Nagaland, Chhattisgarh, Uttar Pradesh and Jammu and Kashmir between 2005 and 06 and 2015-16.

### Decomposition analysis

We carried out the decomposition analysis to capture the contribution of individual covariates. Specifically, both relative and absolute contribution for each of the factor has been gleaned. The sign of the contributing factor could either be positive or negative. A negative value indicates a negative contribution indicating the extent to which a particular variable has increased the level of inequity. On the other hand, a positive value reflects a positive contribution, i.e., the extent to which a particular variable has been responsible for the reduction of level of inequity. Relative and absolute contribution is divulged for 2005-06 as well as 2015-16 to ascertain the pattern of a particular variable in both years.

In this analysis, we found that the contribution of legitimate factors was considerably low. On the other hand, the contribution of illegitimate factors is found to be significantly high. These factors are captured by sociodemographic determinants such as religion, caste, age at marriage, number of under- five children, place of residence, media exposure, mother’s education, size of the household, access-related barriers and community education. Decomposition analysis unveils the contribution of each of these variables in a detailed manner. The unexplained gap (captured by residual) in the health inequity could be attributed to other structural factors which are not included in the analysis due to data constraints.

Our key findings are as follows. First, the contribution of illegitimate factors remained high in 2005-06 and 2015-16. In fact, the positive contribution of legitimate factors reduced by around 2 percentage points, while the contribution of illegitimate factors remained almost same. These variations are evident across state-groups too. For instance, in the high-focus grouped states (80.34 percentage points), the contribution of illegitimate factors is found to be exorbitantly high compared to the non-high focus state groups (71.89 percentage points). During the considered time period, the contribution of illegitimate factors reduced in the high focus grouped states while the same rose for the non-high focus group states. Among the legitimate factors, birth order is found to have a substantial impact on health inequity, the impact of which increased over time. Among the illegitimate factors, the contribution of mother’s education, media exposure and community education were pronounced. Between 2005 and 06 and 2015-16, the contribution of access to media increased over time, while that of mother’s education plummeted. We also found that the impact of access related barriers was quite pronounced in the pre-reform period. However, it is important to note that the contribution of these factors reduced in the post reform period (Table 2).

**Table 2:** Contribution of individual covariates towards the Level of Inequity in CMHS

Individual Covariates	India		Non-High Focused Group states		High Focused Group states	
	05–06	15–16	05–06	15–16	05–06	15–16
Age	0.49	0.11	2.45	1.54	0.24	0.28
Birth Order	5.16	3.73	3.68	2.65	3.81	3.43
BMI Status	−0.02	0.11	0.08	−0.02	0.22	−0.01
***Legitimate Factors***	***5.63***	***3.95***	***6.21***	***4.18***	***4.27***	***3.70***
Religion	−0.07	*1.06*	*−0.75*	*1.27*	−0.61	*− 0.61*
Caste	1.68	0.78	2.61	5.20	6.19	2.85
Age at Marriage	7.85	5.05	9.13	7.18	7.73	6.54
Under-Five Children	0.04	0.85	0.04	0.44	−0.30	1.33
Residence	5.03	8.68	2.60	7.38	11.42	8.52
Access to Media	10.75	21.97	9.69	12.22	9.01	21.70
Mother’s Education	32.52	21.02	34.45	28.22	38.14	27.48
Household Size	−0.31	−0.36	−0.27	−0.45	0.13	−0.42
Access Related Barriers	2.87	0.67	1.74	1.34	2.48	0.63
Community Education	18.20	18.89	11.75	9.09	8.39	12.32
***Illegitimate Factors***	***78.57***	***78.62***	***70.97***	***71.89***	***82.57***	***80.34***
Residual	*15.81*	17.43	22.82	23.93	*13.15*	15.95
Total Inequity	100	100	100	100	100	100

Source: Author’s Computation

## Discussion

Our study made an attempt to compute horizontal inequity and Erreygers Corrected Concentration Index in the utilisation of CMHS after 10 years of implementation of NRHM. The results were compared during and 10 years after the implementation of NRHM. Further, the absolute and relative contributions were gleaned by carrying out decomposition analysis. Our findings reported systematic inequities in the utilisation of CMHS. The positive sign of both Erreygers and horizontal inequity in 2005-06 and 2015-16 indicated that the utilisation of CMHS was favouring rich. Although the major objective of NRHM was to reduce inequity in maternal health care service, we found that the magnitude of pro-rich inequity in CMHS rose by 1.2 percentage points between 2005 and 06 and 2015-16. Our results were analogous to other studies conducted in Indian context [19, 29] and other countries such as Bangladesh [30], Malawi [31] where inequality gradients were found to be favouring richer quintile population in comparison to their poorest counterparts. Evidence have surmised that the inequity in the utilisation of social welfare schemes and system level impediments were few of the supply side constrains causing inequity in the utilisation of maternal health care services. Hence, to reduce inequity in the utilisation of CMHS, it is pertinent to strengthen systemic challenges, particularly in the underdeveloped areas.

There was a market variation in inequity amongst the individual maternal health interventions. In 2005-06 and 2015-16, pro-rich inequity in the utilisation of ANC increased, whereas, inequity in the utilisation of SBA and PNC plummeted across the study period. The reduction of pro-rich inequity in the utilisation of SBA and PNC can be attributed to various state and central level programmatic interventions [17, 32, 33]. The greater uptake of these services could be mainly due to the obvious conjecture that implementation of JSY which provides financial benefits to pregnant women and outcome-based incentives for community health workers for promoting delivery and post-delivery care services has been successful in protecting poor and vulnerable population.

Although, provision of maternal health care services is free in the public health system. But, among rural women reaching the health centre becomes a herculean task when there are no financial incentives- transportation costs associated with multiple ANC visits are prohibitive for vulnerable population [34]. Even though the demand side financing (JSY) has been successful in reducing inequity in the utilisation of SBA and PNC, but its entitlements are not sufficient to motivate the continuum of maternal health care services [35]. Previous studies have provided inconclusive evidence with regard to the inequity in the utilisation of ANC services, few have indicated that inequality in the utilisation of ANC have risen over time [32, 33]. While, others have divulged that inequality in the utilisation of ANC services in India reduced between 2005 and 06 and 2015-16 [19]. The variations in the results could be attributed to the differences in the methodologies. We have standardised the need-based factors while [19] has computed inequalities using standardised concentration indices method.

Increase in the value of HI between 2005 and 06 and 2015-16 warrants a discussion on the changes in the contribution of legitimate and illegitimate factors across the time horizon. Our analysis discerned that the utilization of CMHS is predominantly driven by illegitimate factors such as media exposure, mother’s education, place of residence and access. Our results showed that the contribution of illegitimate factors has increased, while, contribution of legitimate factors has plummeted. One possible explanation would be that mother’s education, media exposure and community education reflect the importance of awareness pertaining to benefits of the utilization of CMHS and repercussions of under-utilization of CMHS should be disseminated to pregnant women belonging to lower quintile groups. Undoubtedly, such information is mostly available among rich women while poor women are often found to grapple with the lack of information pertaining to timely utilization of pre-delivery and post-delivery care respectively. It is well known that the richer population holds higher socio-economic positioning, greater levels of education and better access to different mediums of information [36–42]. To address these disparities, an umbrella of schematic interventions was implemented under the NRHM in 2005 and our results claim that health care system in India has not been able to play an effective role in protecting the poorest population from various socio-economic impediments.

The magnitude of pro-rich inequities in non-high focus group states have reduced after the implementation of NRHM- the overall reduction is mainly compounded by southern states namely, Tamil Nadu and Karnataka. This could be could be attributed to the large contributions of these state governments suggesting that government-led initiatives emphasized egalitarian principles. This echoes the findings of [43] who found these states providing equitable access to maternal health care services by implementing interesting schemes such as expansion of delivery and emergency services to 24 h by asserting greater emphasis in lagging districts. Provision of high standard of antenatal care and delivery care at lower cost. In these states, democratic decentralization has played a significant role in shaping variation at the local level. A study conducted in Kerala, ascertained budgetary allocations- giving around 40 percentage of state’s budget control to the local government improved the care and access across different socio-economic dimensions led to an equitable coverage of health care resources [44]. This suggests an urgent need for state-led initiatives and decentralization of health care across districts, emphasizing poorest and more of neglected sections of the states [43–46]. Even-though gamut of nation-wide interventions such as RSBY and JSY were beneficial [35], state-wise variations in the magnitude and differences in inequities in the utilization of CMHS indicates that state-level targeted interventions can pay off. The authors also emphasized that such schemes take considerable time to affect inequity but it would prove to be noteworthy to considerate state-level targeted interventions and democratic decentralization of CMHS [43, 46–48]. Enormous inequity in the utilization of maternal health care services in high focus group states could be attributed by lack of political commitment [47]. A study conducted in Uttar Pradesh indicated that lack of voice in policies due to the issues related to under-representation owing to their limited capacities in accessing resources impeded the distribution of maternal health services among the marginalized women. Despite gathering concerns about maternal health and raising collective representation, their voices had limited impact on policy decisions at state and central levels impacted them [46, 48].

It is important to note that few of the north-eastern states such as Assam and Sikkim did record a reduction in the level of inequality in the utilization of CMHS. This indicates that, despite asserting greater attention in terms of financial incentives, technical assistance, only few states have been able to reduce the level of pro-rich inequity in high-focus group states. It suggests that the poor situation of maternal health in high focus group states is attributed to lack of state-level political commitment [47, 49]. The reduction in the level of inequality in Assam and Sikkim could be attributed to the successful implementation of JSY in these two states [33].

We carried out a decomposition analysis to explain the significant contributors to health inequity by delineating the contribution of legitimate (need-based) and illegitimate factors (non-need-based). Among the legitimate factors, birth order had a substantial impact on health inequity, and its impact increased over time. It could be mainly because an increase in birth order is associated with a greater level of experience and knowledge about the importance of maternal healthcare services [50, 51]. Among the illegitimate factors, the contribution of mother’s education towards the pro-rich inequality in both 2005-06 (32.52%) and 2015-16 (21.02%) was enormous. Our results support the hypothesis that women with higher educational levels are endowed with more resources in terms of cognition, communication and relationship, making them better decision-makers resulting in better utilisation of healthcare resources [52]. Also, they possess more confidence in handling the officials and are willing to travel far to seek maternal health services [7]. Whereas, those with lower educational levels tend to ignore the benefits of healthcare and are likely to underutilise health services [53]. Our findings are in line with the previous studies suggesting a substantial contribution of education in explaining the amount of pro-rich inequity in the utilisation of maternal health services [10, 54, 55]. Our findings contradict to the findings of [56] who revealed that having a secondary/higher education increased the pro-poor inequity in healthcare utilisation.

We also found that access to media had a massive contribution towards the pro-rich inequity. Our analysis supported the hypothesis that compared to poorest women, wealthier women were more privileged to have access to more than one medium of information. Generally, access to media opens avenues to gather more information about the availability of healthcare services and benefits associated with its utilisation. Between 2005 and 06 and 2015-16, the contribution of access to media has increased by three times in high focus group states. Our results were comparable to the findings of [50, 51].

This study also showed that community literacy had a significant contribution towards pro-rich inequity in the utilisation of CMHS It could be possibly because the concentration of illiterate women in the community indicates problems of limited awareness, lower autonomy and higher incidence of child marriages. These factors together correspond to low decision-making capacity related to healthcare access. Our results were consistent with the findings of Singh and others [57] who revealed that prevalence of higher levels of community poverty and lower levels community education is related to lower utilisation of maternal healthcare services. Finally, access related barriers had significant contribution in the pro-rich inequity in the pre-reform period at national level and in high-focus group states, the contribution of these variables reduced in the post reform period indicating that, NRHM had made some contribution in reducing access related barriers in high-focus group states of India.

## Conclusion and policy recommendations

We observed that inequity in the utilization of CMHS increased between 2005 and 06 and 2015-16. Prominent variations were witnessed across interventions and states. In some states, the level of inequity in the utilization of CMHS reduced, while in others it increased. Across interventions, the pro-rich inequity in ANC increased, whereas for SBA and PNC, the pro-rich inequity witnessed a fall. Important contributing factors for pro-rich inequity were access to media exposure, mother’s education and community level education. On the basis of these findings, we suggest following policy recommendations.

The level of inequity increased between 2005 and 06 and 2015-16 calling for some immediate policy recommendations. First, it is important to undertake immediate steps to increase the utilization of ANC services among poor women. Both state and central government might consider expanding financial incentives for availing ANC services. Outreach programs can be upscaled to provide access to adequate ANC services which entails multiple visits to the facility. Democratization of decentralization as adopted by southern states can be followed by other states as well. Greater contribution of exposure to media and mother’s education indicates the crucial role played by dissemination of knowledge and information related to the benefits of CMHS among poor woman. Government might adopt innovative strategies like advertising on local channels, conducting campaigns and folk shows at village level to spread awareness. Finally, the role of ASHA worker can be strengthened by providing competitive wages and providing proper training facilities to them.

Inequity in the utilization of choice of provider for continuum of maternal health care services is more likely to provide deeper insights into it, but we were unable to estimate that, as it requires information about the range of provider choice which is not captured in NFHS dataset.

## Supplementary Information

**Additional file 1: Table A.1.** Changes in EI values (2005-06 and 2015-16).

## Data Availability

The dataset analysed during the current study are available in the DHS Program website, https://dhsprogram.com/data/available-datasets.cfm.

## Abbreviations

ANC: Antenatal care services
ASHA: Accredited Social Health Activists
BMI: Body Mass Index
CEB: Census enumeration blocks
CC: Concentration Curves
CMHS: Continuum of Maternal Healthcare Services
HI: Horizontal Inequity
IIPS: Institute of Population Sciences
JSY: Janani Suraksha Yojana
MDGs: Millennium Development Goals
MMR: Maternal Mortality Rate
NFHS: National Family Health Survey
NHM: National Health Mission
NRHM: National Rural Health Mission
PNC: Post-natal care services
PPS: Probability Population Size
SBA: Skilled birth attendant
SDGs: Sustainable Development Goals

## Footnotes

1 The states were grouped according to the National Rural Health Mission criteria. *High focus states* encompasses: *major high focus states* (Bihar, Chhattisgarh, Himachal Pradesh, Jammu & Kashmir, Jharkhand, Madhya Pradesh, Odisha, Rajasthan, Uttar Pradesh, and Uttarakhand) and *high focus North-East states* (Arunachal Pradesh, Assam, Manipur, Meghalaya, Mizoram, Nagaland, Sikkim, Tripura).

2 *Non-high focus states* are Andhra Pradesh, Goa, Gujarat, Haryana, Karnataka, Kerala, Maharashtra, Punjab, Tamil Nadu, West Bengal, and Telangana.

3 Illegitimate inequality refers to the amount of inequality that is ethically unacceptable [47; 48] and legitimate inequality refers to the amount of inequality that is considered to be ethically fair, as they emerge due to differences in the biological need.

4 There are eight questions asked in survey related to accessibility barriers preventing women from accessing medical treatment for themselves. The questions are divided into three categories: availability, affordability and acceptability. The response to ‘getting the money needed for treatment’ is categorized as the affordability barrier. The questions on 1) ‘Distance to health facility’, 2) ‘having to take transportation’, 3) ‘concern that there may not be any health provider’, and 4) ‘concern that there may be no drugs available’ are categorized as the availability barrier. Responses to 1) ‘getting permission to go’, 2) ‘not wanting to go alone’, and 3) ‘concern no female health provider’ are categorized as the acceptability barrier.

5 Community level education is defined as the proportion of mothers in the community who had at least a primary level of education
