# Supplementary material for: Horizontal inequity in the utilisation of Continuum of Maternal Health care Services (CMHS) in India: an investigation of ten years of National Rural Health Mission (NRHM)
Source: Int J Equity Health. 2022 Jan 15;21:7. doi: 10.1186/s12939-021-01602-3 (PMC8760767; doi:10.1186/s12939-021-01602-3)
Supplement: Supplementary file 1 — Additional file 1: Table A.1. Changes in EI values (2005-06 and 2015-16). [file 12939_2021_1602_MOESM1_ESM.docx]

**Additional file**

**Table A.1: Changes in EI values (2005-06 and 2015-16)**

| **States of India** | **EI (2005)** | **EI (2015)** | **Difference** | **Direction of Change** |
| --- | --- | --- | --- | --- |
| Andhra Pradesh | 22.17 | 14.42 | 7.75 | DECREASED |
| Arunachal Pradesh | 10.84 | 4.26 | 6.58 | DECREASED |
| Assam | 8.28 | 18.86 | -10.58 | INCREASED |
| Bihar | 7.83 | 4.53 | 3.3 | DECREASED |
| Chhattisgarh | 10.2 | 15.24 | -5.04 | INCREASED |
| Goa | 25.3 | 1.31 | 23.99 | DECREASED |
| Gujarat | 23.77 | 20.52 | 3.25 | DECREASED |
| Haryana | 15.53 | 11.55 | 3.98 | DECREASED |
| Himachal Pradesh | 14.39 | 19.82 | -5.43 | INCREASED |
| Jammu and Kashmir | 17.07 | 22.57 | -5.5 | INCREASED |
| Jharkhand | 8.11 | 11.2 | -3.09 | INCREASED |
| Karnataka | 32.97 | 5.91 | 27.06 | DECREASED |
| Kerala | 10.51 | 5.39 | 5.12 | DECREASED |
| Madhya Pradesh | 9.51 | 12.79 | -3.28 | INCREASED |
| Maharashtra | 18.47 | 15.04 | 3.43 | DECREASED |
| Manipur | 6.78 | 36.75 | -29.97 | INCREASED |
| Meghalaya | 8.59 | 21.7 | -13.11 | INCREASED |
| Mizoram | 13.24 | 30.2 | -16.96 | INCREASED |
| Nagaland | 0.65 | 4.15 | -3.5 | INCREASED |
| Odisha | 19.71 | 8.83 | 10.88 | DECREASED |
| Punjab | 14.64 | 13.97 | 0.67 | DECREASED |
| Rajasthan | 13.8 | 11.05 | 2.75 | DECREASED |
| Sikkim | 25.83 | 0.85 | 24.98 | DECREASED |
| Tamil Nadu | 26.2 | 3.8 | 22.4 | DECREASED |
| Telangana | NA | 14.93 | NA | NA |
| Tripura | 9.38 | 4.77 | 4.61 | DECREASED |
| Uttar Pradesh | 5.32 | 11.02 | -5.7 | INCREASED |
| Uttarakhand | 24.27 | 13.91 | 10.36 | DECREASED |
| West Bengal | 18.14 | 11.53 | 6.61 | DECREASED |
